# The complete genome sequence of Penaeus vannamei nudivirus (previously Baculovirus penaei or P. vannamei singly enveloped nuclear polyhedrosis virus)

**DOI:** 10.1099/mgen.0.001360

**Published:** 2025-02-26

**Authors:** Hung N. Mai, Arun K. Dhar

**Affiliations:** 1 Aquaculture Pathology Laboratory, School of Animal & Comparative Biomedical Sciences, The University of Arizona, 1117 E Lowell St, Tucson, AZ 85721, USA

**Keywords:** BP, nudivirus, *Penaeus vannamei*, PvSNPV

## Abstract

*Penaeus vannamei* singly enveloped nuclear polyhedrosis virus (PvSNPV), also known as Baculovirus penaei (BP), is the first viral pathogen of penaeid shrimp described in 1974. Although PvSNPV was discovered almost 50 years ago, the complete genome sequence has not been elucidated until now. We detected the virus in a quarantine stock of *P. vannamei* shrimp by light microscopy of faecal samples and by PCR screening of broodstock. Subsequently, next-generation sequencing was deployed to determine the complete genome sequence of PvSNPV. The PvSNPV genome is a circular, double-stranded DNA molecule of 119 883 bp in length encoding 101 ORFs. The deduced aa sequences from 28 ORFs were homologous to 28 core proteins from all identified nudiviruses. Phylogenetic analyses based on deduced aa sequences of the core genes and orthologous genes revealed that PvSNPV clusters with *Penaeus monodon* nudivirus. Therefore, we propose to rename BP/PvSNPV as *P. vannamei* nudivirus and re-assign the virus to the family *Nudiviridae* instead of *Baculoviridae*.

**Impact Statement**

Shrimp aquaculture has gained global attention in recent years as a major sector of agriculture and fishery. Currently, infectious diseases remain a bottleneck in the growth and substantiality of this sector. This manuscript describes the genome sequence and evolutionary relation of the very first virus, Baculovirus penaei (BP), reported in marine shrimp about 50 years ago. The virus was tentatively classified as a baculovirus based on morphology. However, genomic architecture and phylogenetic analysis revealed the virus is similar to nudiviruses. Until a few years ago, BP was listed in the World Organization for Animal Health (Paris, France) list of crustacean diseases. The development of management strategies enabled us to reduce the negative impact of the virus in recent years, but it remains prevalent in the hatchery. The availability of the BP genome sequence will be useful in studying host-virus interactions at a molecular level and developing strategies to mitigate the negative impact in the captive breeding programme to develop Specific Pathogen Free lines of shrimp. The availability of the genome sequence of the very first virus reported in penaeid shrimp is an important historical milestone and scientific progress in crustacean research.

## Data Summary

The authors confirm all supporting data, code and protocols have been provided within the article or through supplementary data files.

## Introduction

Commercial shrimp farming started in Asia in 1970 and has grown steadily, reaching an estimated production of 4000 metric tons in 2019 to meet a growing market demand [1]. With the expansion of shrimp aquaculture, infectious diseases became more problematic, and the list of identified shrimp diseases continues to grow in parallel with the growing global shrimp industry [2,3].

Tetrahedral baculovirus, commonly known as Baculovirus penaei (BP) or *Penaeus vannamei* singly enveloped nuclear polyhedrosis virus (PvSNPV), was first reported in the hepatopancreas of pink shrimp, *Penaeus duorarum*, in 1974 in the Gulf of Mexico [4,5]. Later, PvSNPV was reported to cause infections in most penaeid shrimp and during all life stages [6]. The World Organization for Animal Health (WOAH) (OIE, Paris, France) listed PvSNPV as a notifiable crustacean viral pathogen until 2009. In 2009, PvSNPV was delisted because the economic impacts caused by PvSNPV were not as significant as in the past, due to the adoption of simple disease management and biosecurity tools such as washing eggs with iodine-containing water, separating stronger post-larvae (PL) from those that are relatively weaker and quarantining of incoming PLs. Morphologically, the virions are rod-shaped, enveloped particles measuring 312 to 320 nm in length and 75 to 87 nm in diameter. The nucleocapsids, 306 to 312 nm in length and 62 to 68 nm in diameter, have a crosshatched surface arranged in a helical pattern and a trilaminar structure capping both extremities in the virion [7]. The viral particles are present both free and within pyramidal-shaped polyhedral occlusion bodies in the nuclei of hepatopancreatic and midgut cells [5]. The virus was classified as baculovirus because of the morphological similarity of the mature virions to viruses belonging to the family *Baculoviridae*. Viruses in the *Baculoviridae* are the most common insect pathogenic viruses with 4 genera and 97 species [8]. In addition, baculoviruses are well-known as vectors for the transduction of mammalian cells, gene expression vectors and pest control treatments [9,11]. Transmission electron microscope (TEM) analyses revealed that baculoviruses had rod-shaped nucleocapsids surrounded by an envelope [12,13]. Interestingly, in the study by Couch from [4], the author reported that there are differences in lattice line-to-line spacing between PvSNPV and insect-infecting baculoviruses, which led to the early assumption that PvSNPV may not belong to the family *Baculoviridae*.

*Nudiviruses* were a subgroup of *Baculoviridae* before they are officially recognized as an own virus family (*Nudiviridae*) [14]. Nudiviruses are often retrieved from transcriptomic and metagenomic data obtained from invertebrate hosts [15]. *Nudiviridae* is divided into four genera, including *Alphanudivirus*, *Betanudivirus*, *Deltanudivirus* and *Gammanudivirus*. Epsilonnudivirus is proposed as a fifth genus for a divergent group of nudiviruses that infect crustaceans. In addition, *Bracoviriform* is also nominated as a new genus in the *Nudiviridae* family [15,17].

The genera *Alphanudivirus*, *Betanudivirus* and *Deltanudivirus* contain nudiviruses that infect insects, whereas the *Gammanudivirus* and proposed ‘Epsilonnudivirus’ genera have members that infect crustacean species. Recently, there are 12 nudiviruses [i.e. *Aratus pisonii* nudivirus (ApNV), *Crangon crangon* nudivirus (CcNV), *Carcinus maenas* nudivirus (CmNV), *Callinectes sapidus* nudivirus (CsNV), *Dikerogammarus haemobaphes* nudivirus (DhNV), *Faxonius propinquus* nudivirus (FpNV), *Faxonius rusticus* nudivirus (FrNV), *Faxonius virilis* nudivirus (FvNV), *Homarus gammarus* nudivirus (HgNV), *Menippe mercenaria* nudivirus (MmNV), *Macrobrachium rosenbergii* nudivirus (MrNuV) and *Penaeus monodon* nudivirus (PmNV) have been reported in aquatic crustacean species] [17,21]. CsNV, PmNV and PvSNPV were classified as baculovirus, but PmNV and CsNV belong to *Gammanudivirus* [17], suggesting that PvSNPV may belong to *Gammanudivirus*.

The pathobiology of PvSNPV has only been investigated in only a few studies. It is widely accepted that mucosal epithelial cells of the hepatopancreas tubules and anterior midgut from *P. vannamei* are the targets of PvSNPV [6]. Once PvSNPV enters the cells, it creates a highly organized membranous system in the cytoplasm, called membranous labyrinth (ML). Couch [22] hypothesizes that after integrating into the nucleus of the host cell and genetically controlling the membrane-bound cytocavitary system, the PvSNPV elicits the formation of ML through membrane proliferation to construct a transport system (ML cisternae) for transporting the viral structural precursor from the cytoplasm to the nucleus where virion and occlusion bodies are assembled. Since the viral protein is transported from cytoplasm to nucleus for viral reproduction, such active transports need energy to be carried out. In PvSNPV-infected cells, the energy provided for viral component transport is ATP, which is hypothetically obtained from the host cells through the ML membrane, which encircles or occludes proximal mitochondria. Apart from viral component transportation, ML also plays a role in the degeneration of host cell, since ML becomes disorganized at the late point in the viral reproductive cycle, resulting in the collapse of the cell because ML has the large mass in the cytoplasm. In 1988, the first successful PvSNPV experimental trial was done by Overstreet *et al.* who infected *P. vannamei* PL 3 or mysis using *Brachionus plicatilis* and *Artemia salina* as the viral delivery vehicle [23]. Additionally, it was reported that the non-occluded viral particles could provoke infection in *P. vannamei* PL 10 days post-infection [24].

While the pathobiology of PvSNPV was studied [5,22, 24], during the ensuing 45 years, the PvSNPV genome has not been sequenced. Before this study, the largest sequence of PvSNPV deposited in NCBI was less than 2 kbp region containing the polyhedrin gene sequence (DQ496179). Due to this lack of the full genome sequence, the taxonomy of the virus remained unclear, and the molecular basis of viral pathogenicity remained obscure.

In this study, next-generation sequencing (NGS) (Illumina HiSeq 2500 System) was employed to determine the genome sequence of PvSNPV from *P. vannamei* broodstocks that tested positive for the virus. The viral genome was then annotated, and the phylogenetic relationship of PvSNPV to other viruses in the families *Baculoviridae* and *Nudiviridae* was evaluated. Based on the genomic features and phylogenetic relations identified in this study, we suggest the renaming of BP/PvSNPV as *P. vannamei* nudivirus (PvNV) and its reassignment to the family *Nudiviridae* instead of *Baculoviridae*. In this paper, we used PvNV instead of PvSNPV as a newly designated name for this virus.

## Methods

### PvNV source

In 2019, 12 broodstock families were sent to the Aquaculture Pathology Laboratory at the University of Arizona for quarantine and disease screening from a country in Latin America. Upon arrival, samples were collected for screening the WOAH-listed crustacean pathogens as well as PvNV, *Enterocytozoon hepatopenaei* (EHP) and PmNV. Briefly, 30 mg of tissues was collected for DNA/RNA extraction. For enteric pathogens [i.e. *Hepatobacter penaei* (NHP-B), PmNV, PvNV, EHP and acute hepatopancreatic necrosis disease-causing *Vibrio parahaemolyticus* (*Vp*_AHPND_)], hepatopancreases were collected for DNA extraction. For systemic pathogens [i.e. white spot syndrome virus (WSSV), decapod penstylhamaparvovirus 1 (IHHNV), Taura syndrome virus (TSV) and infectious myonecrosis virus (IMNV)], pleopods were collected for DNA/RNA extraction. A total of 20 ng of extracted either DNA or RNA was subjected to conventional PCR or real-time PCR for pathogen screening using primers/probes (Table S2, available in the online Supplementary Material) recommended by the WOAH and other published papers [6,25,28]. Five out of 12 samples turned out to be positive with PvNV.

### PvNV challenge test

A total of 30 shrimp (average weight 1.0 g) were stocked into three 90 l tanks (10 shrimp/tank) with aeration and biofilter. The hepatopancreas from broodstock that were tested PvNV positive was dissected, minced into small pieces and fed to animals in two tanks (1.0 g per tank). The animals in the remaining tank were fed with commercial feed and served as a negative control. On the seventh day post-infection, five shrimp and faeces were collected from each tank for PvNV screening by wet mount examination and PCR analysis. PCR protocol for PvNV detection was obtained from the OIE manual [6].

### Wet mount examinations

Sixty microlitres of Modified Mayer’s Hematoxylin (Thermo Scientific, USA) were dropped into freshly collected faecal samples placed on a glass slide. The faecal samples were gently spread prior to being covered using a cover slip and observed under a light microscope (Leica DM500, Germany) for pyramid-like occlusion body identification.

### Genome sequencing

The DNA from hepatopancreases from PvNV-positive broodstock samples was extracted using the DNeasy Blood and Tissue kit (Qiagen, Germany) following the manufacturer’s instructions. A pool (*N*=5) of extracted DNA was sent for NGS using an Illumina HiSeq 2500 System (PE 2×150 bp) (Illumina) at Omega Bioservices, Norcross, GA. A library of DNA samples was generated at Omega Bioservices using the Library Kit, KAPA Hyper prep for whole genome sequencing (Roche).

### Genome assembly

The DNA reads obtained from Omega Bioservices (Omega Bio-tek, Inc, USA) were quality-trimmed using Trimmomatic v.0.36 (illuminaclip: TruSEQ3-PE, leading:3, trailing:3, minlen:36) [20]. Next, the duplicated reads were removed from quality-trimmed reads using Geneious Prime (version 2019, Biomatters, New Zealand) prior to being paired using Geneious Prime (version 2019, Biomatters, New Zealand). Then, the paired reads were mapped to the *P. vannamei* genome (PRJNA438564) to remove shrimp genome regions using Geneious Prime (version 2019, Biomatters Ltd, New Zealand) (low sensitivities/fastest). The unused reads were assembled *de novo* using the Geneious assembler (medium-low sensitivity/fast) (Geneious Prime v 2019, Biomatters Ltd, New Zealand). A blastn search was carried out with the queries as contigs generated from *de novo* assembly to identify the PvNV contig.

### Genome annotation

The PvNV contig was subjected to annotation. Firstly, the ORFs were predicted using Prokka [29] (default, kingdom=virus) via the Galaxy server (https://usegalaxy.org/), GeneMarks [30,31] (http://exon.gatech.edu/GeneMark/heuristic_gmhmmp.cgi, Virus, genetic code:11) and fgeneV0 (http://www.softberry.com/, standard code, circular sequence). The ORFs were accepted if they were in agreement with at least two of these programs. Secondly, the deduced aa sequences from accepted ORFs were annotated by blastp (blast.ncbi.nlm.nih.gov) sequence similarity searches using the non-redundant protein sequence database (3 October 2024) (*E*-value<10^−13^; coverage>60%) and the PmNV protein database (default setting). Finally, the tandem repeats were found using Tandem Repeat Finder (https://tandem.bu.edu/trf/output/127ZAv7Pv8JVZ.2.7.7.80.10.100.500.1.html, default, score>100). The genome map of PvNV was built using Geneious Prime (version 2019, Biomatters, New Zealand).

### Orthologous and core gene analyses

The putative protein sequences of 15 nudiviruses, 5 baculoviruses and PvNV were subjected to OrthoFinder v2.5.4 (parameter: -A mafft, -M dendroblast, -T fasttree) for identification of orthologous group (Table S4). Paralogous proteins were removed from the orthologous group. Then, the orthologous proteins in each species were concatenated in the same order using Geneious Prime (version 2019, Biomatters, New Zealand) prior to phylogenetic analysis.

In addition, the shared core genes among PvNV and nudiviral species were identified using a blastp search, orthologue analysis and information on core genes of nudivirus from ICTV, 2020 [16]. The shared core genes in each nudiviral species were concatenated in the same order using Geneious Prime (version 2019, Biomatters, New Zealand) prior to phylogenetic analysis.

### Phylogenetic analyses

The concatenation of 19 core/orthologous protein sequences were aligned using MAFFT (select an appropriate strategy according to data size) [32,33] through Geneious Prime (version 2019, Biomatters, New Zealand). The alignment was then trimmed using ClipKIT (-m kpi-gappy) [34] before phylogenetic analysis employing MrBayes 3.2.6 (GTR, Poisson, Gamma) (Markov Chain Monte Carlo parameters: number of generation=10 000; sample a tree every ten generations)[35].

### Syntenic gene analysis

A circle presenting the orientation of the PmNV and PvNV genomes was built by the Circa program (https://omgenomics.com/circa). To investigate if the core genes and orthologue genes between PmNV and PvNV share the same order, the core genes and orthologue genes between PmNV and PvNV were connected by red ribbons or red lines. The parallel patterns made of red ribbons/red lines indicated the same order between PmNV and PvNV orthologue/core genes.

### Polyhedrin promoter analyses

Two hundred nts upstream of the transcription initiation site of polyhedrin genes of two nudiviruses [i.e. PmNV (ORF1) and *Tipula oleracea* nudivirus (ToNV) (ORF59)] and PvNV (ORF96) were subjected to the Neural Network Promoter Prediction (NNPP) server (https://www.fruitfly.org/seq_tools/promoter.html) (type of organism: prokaryote; include reverse strand: no; minimum promoter score: 0.9). The predicted promoter sequences were subjected to Clustal Omega multiple alignment via Geneious Prime (v2023, Biomatter Ltd, New Zealand). The pattern of the polyhedrin promoter was generated by WebLogo (Geneious Prime v2023, Biomatter Ltd, New Zealand).

## Results

### Detection of PvNV in broodstock and experimental challenge of *P. vannamei* PL with PvNV

**Fig. 1.**
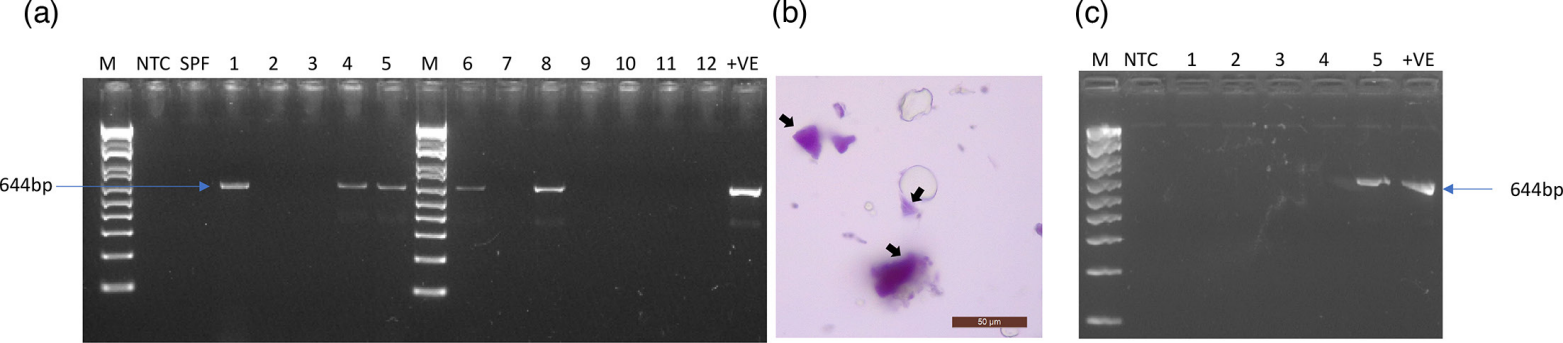
PvNV detection by conventional PCR and wet mount analyses. (**a**) PvNV PCR detection in *P. vannamei* broodstock. M=DNA ladder; NTC=non-template control; SPF=Specific Pathogen Free (SPF) sample; 1–12=quarantine samples; +VE=positive control. (**b**) Wet mount examination of faecal samples originating from SPF *P. vannamei* juvenile experimentally challenged with PvNV. Black arrows indicated occlusion bodies. (**c**) PvNV detection in experimentally PvNV-challenged juveniles. 1–5=PvNV-challenged samples. Blue arrows indicated 644 bp amplicons.

*P. vannamei* broodstock animals were maintained in a quarantine facility for a nucleus breeding programme in the Aquaculture Pathology Laboratory at the University of Arizona. As a part of a routine health check, broodstocks were screened for a panel of enteric and systemic viral, bacterial and fungal pathogens. PvNV was one of the pathogens that was tested using a conventional PCR method using faecal DNA samples. Five out of 12 broodstock tested positive for PvNV (Fig. 1). However, these broodstocks tested negative for WSSV, *Vp*_AHPND_, IHHNV, NHP-B, IMNV, TSV, yellow head virus genotype 1 and EHP.

The PvNV-positive broodstocks were dissected, and hepatopancreas tissue was taken to conduct an experimental bioassay using SPF *P. vannamei* juvenile, as described in the Methods section. A wet mount from faeces from experimentally challenged shrimp displayed ‘pyramid’-like occlusion bodies (Fig. 1). Subsequently, conventional PCR was performed to detect PvNV in the juvenile. One out of five shrimp from the challenged tanks tested positive for PvNV (Fig. 1c).

### *De novo* assembly of PvNV

**Fig. 2.**
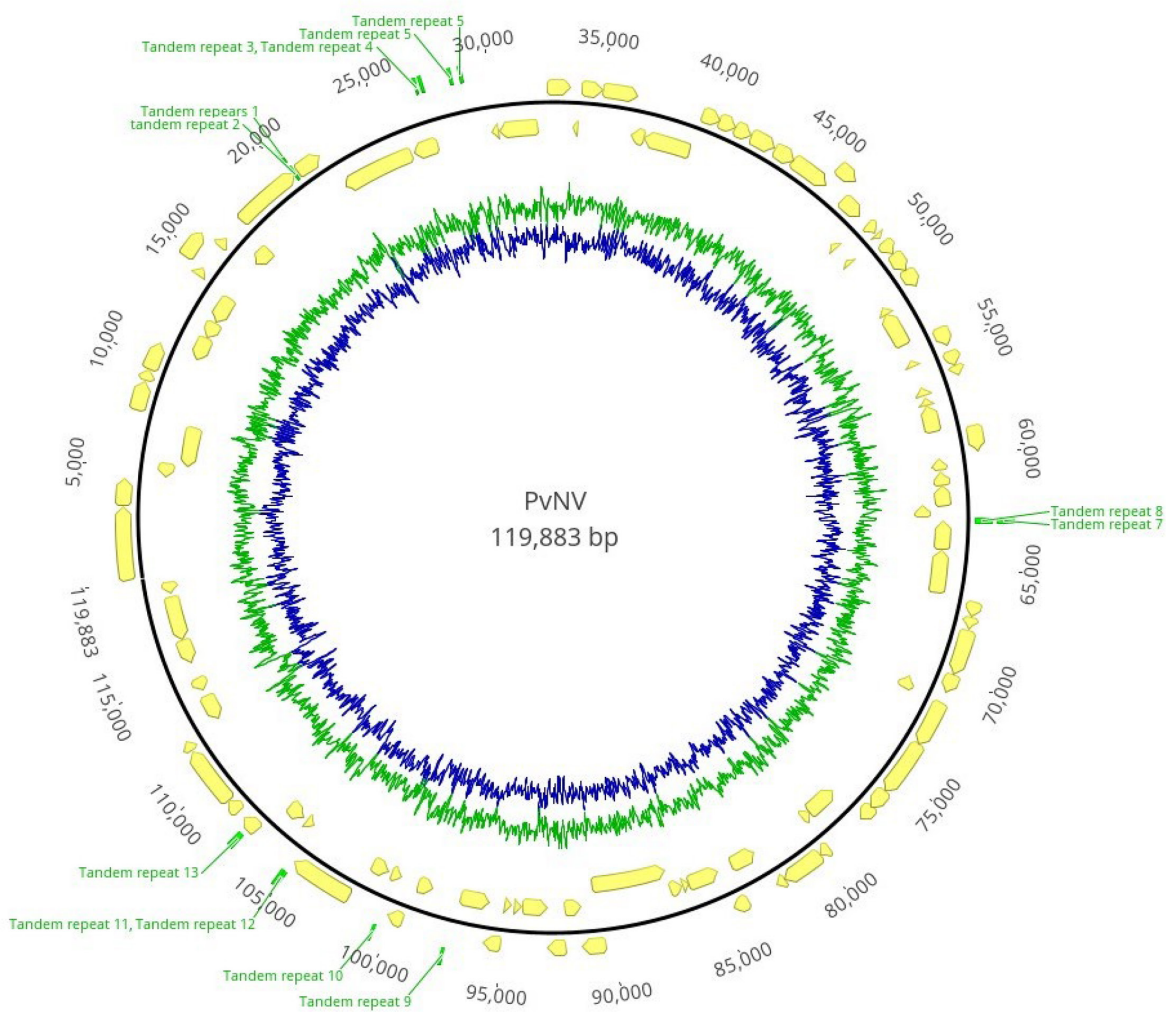
Circular genomic map of *Penaeus vannamei* singly enveloped nuclear polyhedrosis virus (PvNV). The green bars indicate tandem repeat regions. The predicted protein-coding genes and their orientation were indicated by yellow arrows. The outer yellow arrows indicate the predicted protein-coding genes on the forward strand. The inner yellow arrows indicate the predicted protein-coding genes on the reverse strand. The G+C contents are indicated by blue, and the AT contents are indicated by green. The numbers represent the base numbers.

Illumina sequencing generated 16 743 458 unpaired reads. The number of paired reads after adapter, low-quality and duplicated read removals was 10 833 122 paired reads. A total of 9 408 536 paired reads were used to conduct *de novo* assembly after *P. vannamei* genome elimination. *De novo* assembly results showed that 7 224 141 reads were assembled to produce 1 659 960 contigs in which 8521 contigs were longer than 1000 bp in length with a N50=1900 bp. One completely circular contig with 119 883 bp in length was identified in the assembly. In addition, this circular contig showed similarity to the PvSNPV partial sequence available in the GenBank database by blastn (highly similar sequences) search. PvNV contig was assembled by 186 525 paired reads with 234.4× of mean coverage and 33.4% of G+C content (Fig. 2). The PvNV genome was deposited in GenBank with accession number OM066077.

### Characterization of PvSNPV genome

#### ORF prediction

Based on the results from three programs (Prokka, GeneMarks and fgeneV0), 101 ORFs ranging from 201 to 3800 bp in length were annotated (Table 1) and mapped on the genome (Fig. 2). In addition, the number of annotated ORFs was equal between forward and reverse strands (51 ORFs in the forward strand; 50 ORFs in the reverse strand). The gene density was ~0.8 gene per kbp.

**Table 1.** Identified ORF in the PvSNPV genome. The start and stop positions were shown, as well as the length (in nt and aa) and the strand (‘+’: forward strand; ‘−’: reverse strand). The best hits obtained using blastp (protein-protein blast) using a non-redundant protein sequence (nr) database on NCBI (3 October 2024) (*E*-value-threshold=10^−13^, coverage=60%). Additional information was obtained from orthologous analysis. The core genes were bolded

ORF	Start	Stop	Length		Strand	Best hit (name/description/accession no.)	%id	Coverage%	*E*-value	Additional annotation
			**nt**	aa						
**1**	1	3306	3306	1101	+	**DNA polymerase** (*Penaeus monodon* nudivirus) (YP_009051843.1)	59.89	98	0	**DNApol**
**2**	3347	4561	1215	404	+	Methyltransferase (*Penaeus monodon* nudivirus) (YP_009051844.1)	43.92	98	1×10^−105^	
**3**	4776	5447	672	223	−	**Ac92-like protein** (*Penaeus monodon* nudivirus) (YP_009051846.1)	53.43	91	3×10^−72^	**P33**
**4**	5425	7467	2043	680	+	**VP91** (*Penaeus monodon* nudivirus) (YP_009051847.1)	48.15	98	0	**P95**
**5**	7603	8925	1323	440	−	**ODV-E56** (*Penaeus monodon* nudivirus) (YP_009051848.1)	57.01	96	1×10^−176^	**PIF-5**
**6**	9005	9484	480	159	−	Hypothetical protein PmNV-012 (*Penaeus monodon* nudivirus) (YP_009051850.1)	40.25	98	2×10^−33^	
**7**	9494	10 777	1284	427	+	**P47** (*Penaeus monodon* nudivirus) (YP_009051852.1)	50.83	97	7×10^−151^	
**8**	10 782	11 912	1131	376	+	**PIF-2** (*Penaeus monodon* nudivirus) (YP_009051853.1)	61.6	98	6×10^−171^	
**9**	11 955	12 746	792	263	−	HZV-115-like protein (*Penaeus monodon* nudivirus) (YP_009051855.1)	32.77	82	4×10^−34^	
**10**	12 834	14 183	1350	449	−	Hypothetical protein PmNV-018 (*Penaeus monodon* nudivirus) (YP_009051856.1)	34.19	91	2×10^−63^	
**11**	14 207	14 587	381	126	+	Hypothetical protein PmNV-019 (*Penaeus monodon* nudivirus) (YP_009051857.1)	45.67	96	1×10^−24^	
**12**	14 545	15 813	1269	422	+	Hypothetical protein PmNV-020 (*Penaeus monodon* nudivirus) (YP_009051858.1)	45.52	99	4×10^−114^	**FEN-1**
**13**	15 800	16 273	474	157	−	Hypothetical protein PmNV-021 (*Penaeus monodon* nudivirus) (YP_009051859.1)	37.34	100	5×10^−27^	
**14**	16 282	17 202	921	306	−	Hypothetical protein PmNV-022 (*Penaeus monodon* nudivirus) (YP_009051860.1)	58.58	99	3×10^−130^	**31 k/VP39**
**15**	17 320	20 415	3096	1031	−	**LEF8** (*Penaeus monodon* nudivirus) (YP_009051861.1)	57.23	100	0	
**16**	20 595	21 818	1224	407	−	Hypothetical protein (*Penaeus monodon* nucleopolyhedrovirus) (ABO38811.1)	52.81	99	1×10^−139^	P51
**17**	22 084	25 659	3576	1191	+	Hypothetical protein PmNV-025 (*Penaeus monodon* nudivirus) (YP_009051863.1)	42.74	51	2×10^−146^	
**18**	25 813	27 027	1215	404	−	None				
**19**	29 758	30 054	297	98	+	None				
**20**	30 117	32 012	1896	631	−	ODV-E66 (*Penaeus monodon* nudivirus) (YP_009051874.1)	54.76	99	0	
**21**	32 538	33 569	1032	343	−	None				
**22**	33 745	33 984	240	79	−	None				
**23**	34 049	34 978	930	309	+	Guanosine monophosphate kinase (*Penaeus monodon* nudivirus) (YP_009051876.1)	47.78	100	7×10^−88^	TK2
**24**	35 027	36 598	1572	523	+	**PIF-1** (*Penaeus monodon* nudivirus) (YP_009051877.1)	52.85	99	0	
**25**	36 623	37 264	642	213	−	None				
**26**	37 267	39 576	2310	769	+	Hypothetical protein PmNV_042 (*Penaeus monodon* nudivirus) (YP_009051880.1)	44.53	82	1×10^−162^	
**27**	39 567	40 367	801	266	−	Hypothetical protein PmNV_043 (*Penaeus monodon* nudivirus) (YP_009051881.1)	50.61	91	4×10^−86^	
**28**	40 386	41 126	741	246	−	Hypothetical protein PmNV_044 (*Penaeus monodon* nudivirus) (YP_009051882.1)	27.78	93	3×10^−17^	
**29**	41 126	41 890	765	254	+	PmV-like protein (*Penaeus monodon* nudivirus) (YP_009051883.1)	56.68	95	5×10^−96^	
**30**	41 938	43 074	1137	378	+	p-Loop NTPase (*Penaeus monodon* nudivirus) (YP_009051884.1)	48.22	88	1×10^−101^	TK3
**31**	43 142	44 062	921	306	+	Hypothetical protein PmNV_047 (*Penaeus monodon* nudivirus) (YP_009051885.1)	45.54	99	2×10^−82^	
**32**	44 067	45 863	1797	598	−	Hypothetical protein PmNV_048 (*Penaeus monodon* nudivirus) (YP_009051886.1)	49.75	97	0	
**33**	45 842	46 747	906	301	+	None				
**34**	46 907	47 923	1017	338	+	None				
**35**	47 925	48 248	324	107	+	Hypothetical protein (*Penaeus mondon* nudivirus) (ABX44699)	42.72	96	4×10^−15^	
**36**	48 455	48 943	489	162	+	**LEF-5** (*Callinectes sapidus* nudivirus) (UVX94904.1)	32.7	95	4×10^−17^	
**37**	49 005	49 268	264	87	+	None				
**38**	49 478	50 137	660	219	+	Hypothetical protein PmNV_054 (*Penaeus monodon* nudivirus) (YP_009051892.1)	45.91	99	1×10^−54^	
**39**	50 178	51 089	912	303	−	**Integrase** (*Penaeus monodon* nudivirus) (YP_009051893.1)	61.06	100	3×10^−138^	
**40**	51 082	51 951	870	289	−	Hypothetical protein (*Penaeus mondon* nudivirus) (ABX44703)	52.07	99	3×10^−87^	**VLF1a**
**41**	51 941	52 273	333	110	−	None				
**42**	52 263	54 011	1749	582	−	**LEF-9** (*Penaeus monodon* nudivirus) (YP_009051896.1)	61.76	89	0	
**43**	54 029	54 874	846	281	+	**38K** protein (*Penaeus monodon* nudivirus) (YP_009051897.1)	50	100	5×10^−85^	
**44**	54 883	55 167	285	94	−	Hypothetical protein PmNV_060 (*Penaeus monodon* nudivirus) (YP_009051898.1)	52.13	100	4×10^−23^	
**45**	55 169	55 861	693	230	−	Hypothetical protein PmNV_061 (*Penaeus monodon* nudivirus) (YP_009051899.1)	54.74	100	1×10^−80^	
**46**	55 863	56 276	414	137	−	Hypothetical protein PmNV_062 (*Macrobrachium rosenbergii* nudivirus) (YP_009051900.1)	27.94	99	1×10^−16^	**GbNV_GP51**
**47**	56 289	56 681	393	130	−	None				
**48**	56 821	57 174	354	117	+	None				
**49**	57 238	58 557	1320	439	+	p-Loop NTPase (*Penaeus monodon* nudivirus) (YP_009051903.1)	49.55	100	2×10^−158^	TK1
**50**	58 623	59 804	1182	393	−	Hypothetical protein PmNV_066 (*Penaeus monodon* nudivirus] (YP_009051904.1)	45.34	92	5×10^−109^	
**51**	59 876	60 403	528	175	−	Hypothetical protein PmNV_067 (*Penaeus monodon* nudivirus) (YP_009051905.1)	32.77	94	9×10^−19^	
**52**	60 555	61 166	612	203	−	Hypothetical protein PmNV_068 (*Penaeus monodon* nudivirus) (YP_009051906.1)	40.78	87	1×10^−31^	
**53**	61 196	62 176	981	326	+	Hypothetical protein PmNV_069 (*Penaeus monodon* nudivirus) (YP_009051907.1)	54.68	98	6×10^−114^	
**54**	62 154	62 750	597	198	−	Hypothetical protein PmNV_070 (*Penaeus monodon* nudivirus) (YP_009051908.1)	48.73	96	7×10^−49^	
**55**	62 936	64 315	1380	459	+	Hypothetical protein PmNV_071 (*Penaeus monodon* nudivirus) (YP_009051909.1)	31.36	80	8×10^−53^	
**56**	64 350	66 431	2082	693	+	**P74** (*Penaeus monodon* nudivirus) (YP_009051910.1)	57.56	98	0	**PIF-0**
**57**	66 525	67 217	693	230	+	None				
**58**	67 159	67 769	611	169	+	Hypothetical protein PmNV_075 (*Penaeus monodon* nudivirus) (YP_009051913.1)	39.39	97	9×10^−27^	
**59**	67 852	69 828	1977	658	−	**Helicase 2** (*Penaeus monodon* nudivirus) (YP_009051914.1)	53.62	86	0	**Helicase 2**a
**60**	69 873	70 754	882	293	+	None				
**61**	70 735	71 361	627	208	−	Hypothetical protein PmNV_078 (*Penaeus monodon* nudivirus) (YP_009051916.1)	51.2	78	3×10^−53^	
**62**	71 258	73 339	2082	693	+	**Helicase 2** (*Penaeus monodon* nudivirus) (YP_009051917.1)	49.86	99	0	**Helicase 2b**
**63**	73 332	75 878	2547	848	+	Hypothetical protein PmNV_080 (*Penaeus monodon* nudivirus) (YP_009051918.1)	28.86	96	2×10^−59^	
**64**	75 880	76 827	948	315	+	Hypothetical protein PmNV_082 (*Penaeus monodon* nudivirus) (YP_009051920.1)	45.34	97	5×10^−80^	
**65**	76 818	77 576	759	252	−	None				
**66**	77 573	79 060	1488	495	+	Hypothetical protein PmNV_084 (*Penaeus monodon* nudivirus) (YP_009051922.1)	34.72	96	3×10^−76^	
**67**	79 069	79 479	411	136	+	Hypothetical protein PmNV_085 (*Penaeus monodon* nudivirus) (YP_009051923.1)	42.54	97	2×10^−27^	
**68**	79 460	79 963	504	167	−	**Ac81-like protein** (*Penaeus monodon* nudivirus) (YP_009051924.1)	63.4	91	2×10^−66^	
**69**	79 960	81 879	1920	639	+	Hypothetical protein PmNV_087 (*Penaeus monodon* nudivirus) (YP_009051925.1)	41.6	96	3×10^−156^	
**70**	81 876	82 307	432	143	+	**PIF-6** (*Carcinus maenas* nudivirus) (UBZ25668.1)	47.55	100	2×10^−42^	
**71**	82 415	83 689	1275	424	−	Hypothetical protein PmNV_089 (*Penaeus monodon* nudivirus) (YP_009051927.1)	45.54	97	5×10^−109^	
**72**	83 759	84 469	711	236	+	**VLF-1** (*Penaeus monodon* nudivirus) (YP_009051928.1)	56.17	99	2×10^−79^	**VLF-1b**
**73**	84 471	85 949	1479	492	+	**LEF-4** (*Penaeus monodon* nudivirus) (YP_009051929.1)	48.69	92	7×10^−147^	
**74**	85 964	86 275	312	103	−	None				
**75**	86 326	86 922	597	198	−	**PIF-3** (*Carcinus maenas* nudivirus) (UBZ25673.1)	51.03	96	3×10^−65^	
**76**	86 901	90 710	3810	1269	−	**Helicase** (*Penaeus monodon* nudivirus) (YP_009051932.1)	52.54	99	0	
**77**	90 394	91 458	1065	354	−	**ODV-E28** (*Penaeus monodon* nudivirus) (YP_009051934.1)	51.5	64	3×10^−84^	**PIF-4**
**78**	91 382	92 185	804	267	+	Hypothetical protein PmNV_097 (*Penaeus monodon* nudivirus) (YP_009051935.1)	51	93	3×10^−79^	
**79**	92 175	92 999	825	274	+	**Esterase** (*Penaeus monodon* nudivirus) (YP_009051936.1)	54.87	100	2×10^−105^	**GbNV-gb19-like protein**
**80**	93 003	94 232	1230	409	+	Hypothetical protein PmNV_099 (*Penaeus monodon* nudivirus) (YP_009051937.1)	51.55	46	4×10^−54^	**GbNV-gb67-like protein**
**81**	94 310	94 618	309	102	+	**11K virion structural protein** (*Penaeus monodon* nudivirus) (YP_009051938.1)	59.79	95	4×10^−35^	
**82**	94 739	95 095	357	118	+	None				
**83**	95 082	95 879	798	265	−	Hypothetical protein PmNV_102 (*Penaeus monodon* nudivirus) (YP_009051940.1)	56.23	98	8×10^−100^	
**84**	95 867	97 246	1380	459	−	Hypothetical protein PmNV_103 (*Penaeus monodon* nudivirus) (YP_00951941.1)	30.29	65	8×10^−27^	
**85**	98 733	99 446	714	237	+	Hypothetical protein PmNV_107 (*Penaeus monodon* nudivirus) (YP_009051945.1)	71.55	97	8×10^−123^	
**86**	99 573	100 256	684	227	+	Hypothetical protein PmNV_108 (*Penaeus monodon* nudivirus) (YP_009051946.1)	52.89	99	4×10^−82^	
**87**	100 348	100 887	540	179	−	Baculoviral IAP repeat-containing protein 2 (*Armadillidium vulgare*) (RXG68825.1)	30.67	79	6×10^−14^	IAP2
**88**	101 084	101 866	783	260	−	Inhibitor of apoptosis protein (*Penaeus monodon*) (AIG94824)	39.23	96	4×10^−46^	IAP
**89**	102 157	105 006	2850	949	−	Hypothetical protein PmNV-003 (*Penaeus monodon* nudivirus) (YP_009051841.1)	27.6	95	4×10^−91^	
**90**	105 353	105 763	411	136	+	None				
**91**	106 036	106 866	831	276	+	Baculoviral IAP repeat containing protein 2-like (*Penaeus monodon* majanivirus B) (BDT61976.1)	32.92	78	4×10^−29^	
**92**	107 219	108 046	828	275	+	None				
**93**	108 350	109 000	651	216	−	None				
**94**	109 079	111 745	2667	888	−	Hypothetical protein PmNV-003 (*Penaeus monodon* nudivirus) (YP_009051841.1)	27.01	97	2×10^−78^	
**95**	111 803	112 291	489	162	−	None				
**96**	112 402	113 694	1293	430	+	Polyhedrin (*Penaeus monodon* nudivirus) (ABY75164.1)	49.66	96	2×10^−131^	
**97**	113 956	114 639	684	277	−	None				
**98**	115 484	116 620	1137	378	−	None				
**99**	116 701	118 818	2118	705	+	None				
**100**	118 969	119 511	543	180	+	Uncharacterized protein LOC134780830 (*Penaeus indicus*) (XP_037773819.1)	44.86	59	1×10^−21^	
**101**	49 039	49 239	201	67	+	**Putative p6.9**				

#### Tandem repeats

A total of 13 tandem repeat regions were identified along the genome of PvNV. Three out of 13 tandem repeats partially overlapped two predicted ORFs (Fig. 2) (Table S1). The repeat region length ranged from 60 to 230 bp.

#### Orthologous and core gene analyses

The putative protein sequences from 22 nudiviruses, 5 baculoviruses and PvNV were analysed using OrthoFinder v2.5.4. The results showed that 2526 (78.7%) out of 3211 protein sequences were assigned to 424 orthogroups. Fifty percent of all genes were categorized in orthogroups, with at least eight genes per group (G50=8), and these genes were contained in the largest 120 orthogroups (O50=120). Interestingly, the results showed that PvNV was in 27–80 orthogroups with other nudiviruses. In particular, PvNV was in 80 orthogroups with PmNV (Table 2).

**Table 2.** The species overlap in orthogroups. The number represented the number of orthogroups

The reciprocal blastp and orthologous analyses showed that 28 out of 28 nudivirus core proteins were identified in PvNV (Tables 3, S3). Five ORFs involved in DNA repair and replication (i.e. *dna polymerase*, *integrase*, two *helicase-2* and a *helicase*) were identified in the PvNV genome (ORF1, ORF39, ORF59, ORF62, and ORF76) (Table 1). In addition, orthologous analyses also showed that the deduced aa sequence from ORF12 was in the orthogroup, FEN-1, which is responsible for DNA repair. Like other nudiviruses infecting crustacean species, *helicase-2* was found to be a duplication event gene in the PvNV genome. Four ORFs, which are predicted to be involved in transcription in nudiviruses, were also identified in the PvNV genome. The deduced aa sequences from ORF7, ORF15, ORF42 and ORF73 matched with P47, LEF-8, LEF-9 and LEF-4, respectively (Table 1). Orthologous analyses revealed that the deduced aa sequence from ORF36 was in the LEF-5-annotated orthogroup. The deduced aa sequences from ORF5, ORF8, ORF24, ORF56, ORF70, ORF75, and ORF77 were homologous to ODV-E56, PIF-2, PIF-1, P74, PIF-6, PIF-3 and ODV-E28, respectively (Table 1). Those proteins were well documented to be involved in *per os* infection [36]. The genes involved in viral packaging, assembly and release were also identified in the PvNV genome. For instance, the deduced aas from ORFs 81, 43, 68, 4, 3, 14 and 72 were homologous to the 11K-like, 38K, AC-81, VP91, AC92, VP39 (also called 31K) and VLF-1 proteins, respectively. In addition, by using a manual search, we found p6.9 in the PvNV genome (Table 1).

**Table 3.** Core gene comparison between PvSNPV, nudiviruses and baculoviruses. The ‘*’ indicates the presence of genes

Gene	Description	PvNV	Nudiviruses	Baculoviruses
**DNA replication, repair and transcription**				
*dnapol*	DNA polymerase	*	*	*
*helicase*	DNA helicase	*	*	*
*helicase-2*	DNA helicase	*	*	
*fen-1*	FEN-1/FLAP endonuclease	*	*	
*integrase*	DNA processing	*	*	
*lef-1*	DNA primase			*
*lef-2*	DNA replication/primase-associated factor			*
*gp41*	Tegument protein			*
*alk exo*	DNA recombination and replication			*
*lef-4*	RNA polymerase subunit	*	*	*
*lef-5*	Transcription initiation factor	*	*	*
*lef-8*	RNA polymerase subunit	*	*	*
*lef-9*	RNA polymerase subunit	*	*	*
*p47*	RNA polymerase subunit	*	*	*
**Oral infection, viral packaging, assembling and releasing**				
*pif-0/p74*	*Per os* infectivity factor	*	*	*
*pif-1*	*Per os* infectivity factor	*	*	*
*pif-2*	*Per os* infectivity factor	*	*	*
*pif-3*	*Per os* infectivity factor	*	*	*
*pif-4/odv-e28*	*Per os* infectivity factor	*	*	
*pif-5/odv-e56*	*Per os* infectivity factor	*	*	
*pif-6*	*Per os* infectivity factor	*	*	
*11K-like*	Occlusion body component	*	*	
*38K*	Nucleocapsid protein	*	*	*
*ac81*	Nucleocapsid envelopment	*	*	*
*vp39*	Major capsid protein	*	*	*
*p6.9*	Nucleocapsid packaging/assembly	*	*	*
*vp91/p95*	Nucleocapsid protein	*	*	*
*p33_ac92*	Sulfhydryl oxidase	*	*	*
*vlf-1*	Very late gene expression factor	*	*	*
*ac53*	Nucleocapsid assembly			*
*vp1054*	Nucleocapsid protein			*
*Desmop*	Present in nucleocapsid			*
*p18*	Egress of nucleocapsids			*
*p40*	Subunit of protein complex			*
*odv-e25*	ODV envelope protein			*
*odv-ec43*	Associated with ODV			*
*ac96*	Require for PIF-4			*
*p48*	BV production and ODV envelopment			*
*49* k	Require for BV production			*
**Unknown function**				
*GbNV_gp19-like/esterase*	Unknown	*	*	
*GbNV_gp51-like*	Unknown	*	*	
*GbNV_gp67-like*	Unknown	*	*	
*ac78*	Unknown			*

Three nudiviral core genes with unknown functions were also identified in the PvNV genome. The deduced aa sequence of ORF79 was in the orthogroup with the GBNV-GB19-like protein. Additionally, orthologous analyses revealed that the deduced aa sequence from ORF80 was in the orthogroup with the GBNV-GB67-like protein, which is one of the nudivirus unknown functional core proteins (Table 1). Orthologue analysis revealed that the deduced aa sequence from ORF 46 was in the orthogroup with the GBNV-GB51-like protein.

#### Phylogenetic analyses

**Fig. 3.**
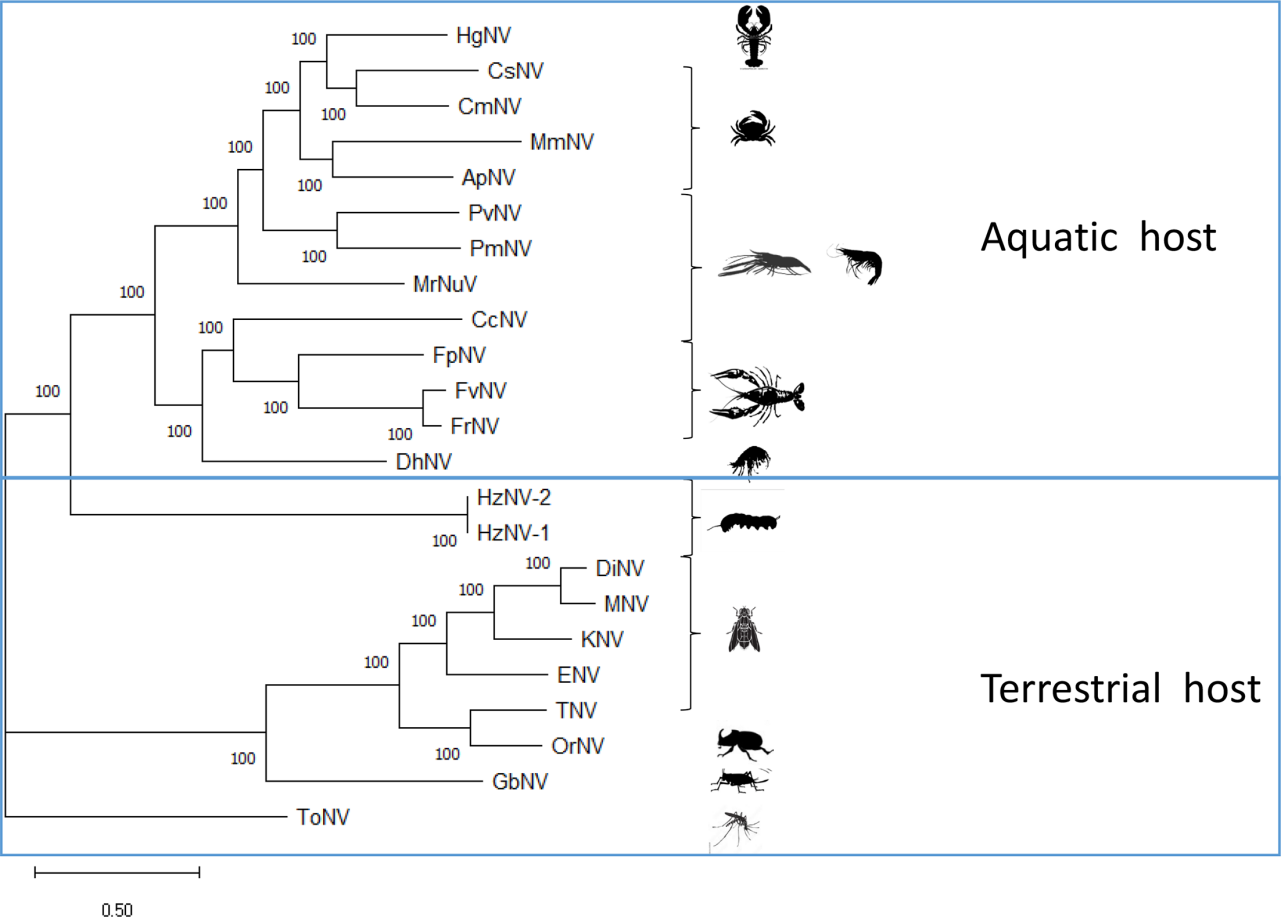
Phylogeny analyses of PvNV and other nudiviruses. Supermatrix phylogenetic tree based on 19 core/orthologous protein sequences (DNA polymerase, helicase, LEF-4, LEF-5, LEF-8, LEF-9, PIF-0/P74, PIF-1, PIF-2, PIF-3, PIF-4, PIF-5, PIF-6, 38K, AC-81, AC-92, VP39 and VP91) from 23 nudiviruses including PmNV (NC_024692), PvNV (OM066077), ApNV (ON061174.1), CcNV (MZ311577), CmNV (MZ311578), CsNV (ON638996.1), DhNV (MT488302.1), *Drosophila innubila* nudivirus (DiNV) (NC_040699.1), Esparto virus (ENV) (NC_040536.1), FpNV (PP539709), FrNV (PP539711), FvNV (PP539710), *Gryllus bimaculatus* nudivirus (GbNV) (NC_009240.1), HgNV (MK439999.1), *Heliothis* nudivirus 1 (HzNV-1) (AF451898.1), *Heliothis *nudivirus 2 (HzNV-2) (NC_004156.2), Kallithea virus (KNV) (NC_033829.1), MmNV (OQ725696.1), Mauternbach virus (MNV) (MG969167), MrNuV (MW484891.1), *Oryctes rhinoceros* nudivirus (OrNV) (NC_011588.1), Tomelloso virus (TNV) (NC_040789.1) and ToNV (NC_026242.1).

The phylogenetic tree based on the concatenation of 19 orthologous/core protein sequences (i.e. DNA polymerase, helicase, LEF-4, LEF-5, LEF-8, LEF-9, PIF-0/P74, PIF-1, PIF-2, PIF-3, PIF-4, PIF-5, PIF-6, 38K, AC-81, AC-92, VP39 and VP91) clustered PvNV with the same clade as PmNV with a posterior probability of 100% (Fig. 3).

#### Syntenic gene analysis

**Fig. 4.**
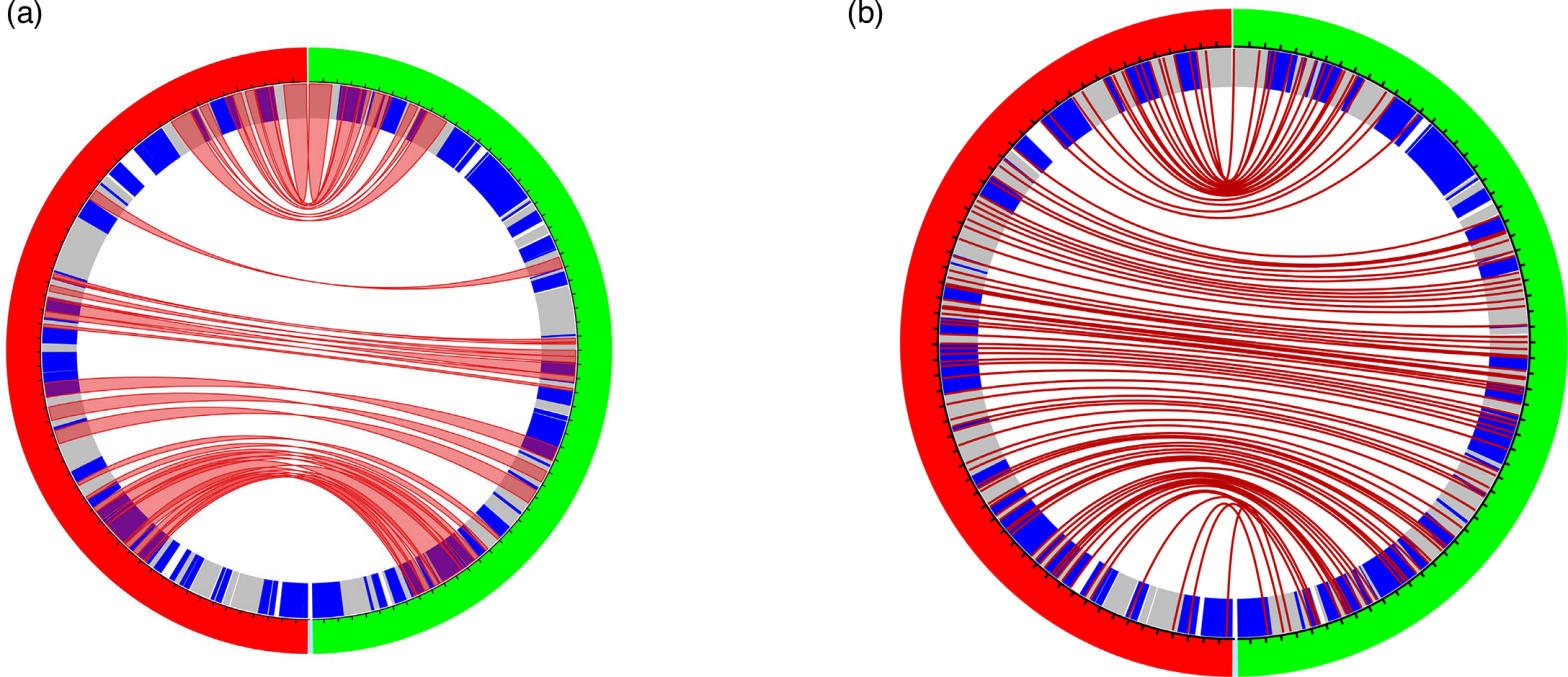
A syntenic gene analysis between PmNV (green) and PvNV (red). (**a**) Syntenic gene analysis of core genes between PmNV and PvNV. The red ribbons connected the core genes between PmNV and PvNV. (**b**) Syntenic gene analysis of orthologue genes between PmNV and PvNV. The red lines connected orthologue genes between PmNV and PvNV. The genes on the forward strand were indicated by grey colour. The genes on the reverse strand were indicated by blue colour. The figure was generated by Circa (https://omgenomics.com/circa/).

The synteny results showed that PmNV and PvNV shared the same arrangement in the order of core genes (Fig. 4).

#### Polyhedrin promoter analysis

The nt sequence upstream of the polyhedrin gene from three nudiviruses, including ToNV (ORF59), PmNV (ORF1) and PvNV (ORF96), was analysed by the NNPP server to search for promoters.

**Fig. 5.**
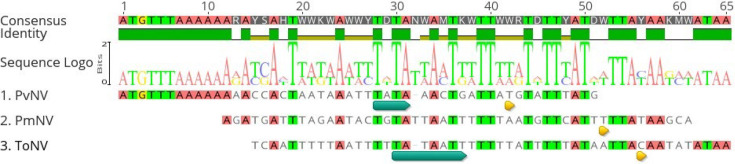
A promoter analysis of polyhedrin gene. Multiple alignment (clustalw) of putative promoters of polyhedrins from three nudiviruses predicted by NNPP (type organism: prokaryote; include reverse strand: no; minimum promoter score: 0.9) (Reese, 2001). The TSS was indicated by yellow arrows, and TATA boxes were indicated by cyan arrows. The sequence logo of polyhedrin alignment was generated by the WebLogo (Geneious Prime v.2023). The higher letters indicated higher levels of consensus.

Putative promoter regions were identified in all three viruses, and the TATA boxes were identified in two out of three putative promoter regions. The TATA boxes were located at −9 to −18 positions. Regarding the polyhedrin promoter from PvNV, the sequence between the transcription start site (TSS) and the start codon of polyhedrin ORF has an A/T rich sequence. Multiple alignments of polyhedrin promoters from three nudiviruses revealed that the polyhedrin promoters contained the consensus sequences AxTxxxxAxxxTxTAxxAxTxxTTxxxTxTTxAT (Fig. 5).

## Discussion

PvNV (also known as BP) and PmNV (also known as monodon baculovirus (MBV) are the first two shrimp viruses identified in 1974 and in 1977 [37], respectively. Based on transmission electron microscope (TEM) images, both MBV and BP were initially identified as viruses belonging to the family *Baculoviridae* due to morphological similarities to other baculoviruses. In addition, the presence of occlusion-derived viruses in BP and MBV was similar to other members of the *Baculoviridae* family that generate occlusion-derived viruses to infect insect species [38]. However, the molecular phylogenetic analysis of six viral genes revealed that the virus called MBV belongs to the *Nudiviridae* family and not to the *Baculoviridae* family, and it is now renamed as PmNV [36,39]. We were interested in determining the nt sequence and examining the taxonomic affiliation of BP (PvNV) at the genome level.

In the present study, PvNV was detected in the *P. vannamei* broodstock held in a quarantine facility during a routine screening. Subsequently, infected broodstocks were sacrificed, and *per os* challenge was performed using SPF *P. vannamei* juvenile to determine the infectivity of the virus detected in the broodstock. When faecal samples of the juvenile were examined by routine light microscopy, pyramid-like polyhedral occlusion bodies that are considered characteristic of PvNV infection were observed. In addition, the PvNV was also detected in the hepatopancreas of the *per os* challenged juvenile by PCR, confirming the infectivity of the virus originating in broodstock in the quarantine facility. However, the infectivity of PvNV in this study was not as high (20% as determined by the PCR-positive test) as previously reported (e.g. PvNV); prevalence is 80% after 6 days of infection [24]. The lower infectivity of PvNV in this study was likely due to the developmental stage of the animals used in the present study (i.e. ~1.0 g size juvenile) compared to PL used in Hammer’s study (i.e. PL 9). In addition, the genetic background of the juvenile used in the present study is very likely different from the one used by Hammer *et al.* [24]. There is a possibility that many genetic lines of commercially available SPF *P. vannamei* shrimp are tolerant/resistant to BP. Despite the relatively lower infectivity, the *per os* experiment confirmed that we were dealing with infectious PvNV. The total genomic DNA obtained from broodstock samples was taken for NGS analysis.

In the ‘80s through ‘90s, hematoxylin and eosin-stained histology, TEM and biophysical properties of viruses were used to classify shrimp viruses [40]. From 2000 onwards, as shrimp viral genomes were sequenced from purified viruses, viral classification was made by combining histopathology, biophysical properties of virions and genome sequence data. In recent years, NGS has been utilized to accelerate viral genome sequencing, and the availability of large sequence data in a relatively short time has further accelerated the use of such data in shrimp virus classification. As a result, shrimp viruses that were previously classified based solely on morphological and histopathological data have been reclassified based on their genomic characteristics. It is for this reason that MBV that was originally classified as a baculovirus is now tentatively assigned as a member of the family *Nudiviridae* [36]. Similarly, the genome sequence data revealed that BP should be reclassified as a member of the family *Nudiviridae*, as described in this study.

We used NGS data to characterize PvNV that was isolated from *P. vannamei* broodstock originating in a Latin American country. The circular genome sequence of PvNV was ~120 kbp, which falls within the range of the genome size of baculoviruses (80–180 kbp) and nudiviruses (97–230 kbp) [39,41]. However, orthologous analysis and blast search using the nr database on NCBI (Oct 03, 2024) revealed that all core genes of nudivirus were found in the PvNV genome sequence. Besides, the *integrase* gene, which is always absent in baculovirus but is a core gene in nudivirus [14,18], was identified in PvNV. This evidence suggested that PvNV should be classified as a member of the family *Nudiviridae*. The members of the *Nudiviridae* family infect several insect and crustacean species, and the virus replicates in the nuclei of host cells [16,17, 19,21]. The virions of nudivirus are made of cylindrical nucleocapsids. Those nucleocapsids are enveloped to produce rod-shaped virions that display a variety of lengths and widths [16]. Initially, the nudiviruses were classified as ‘non-occluded baculoviruses’, due to the similarity in structure sharing between nudiviruses and baculoviruses [42]. Later, nudiviruses were classified as ‘intranuclear bacilliform viruses’ after they were removed from the *Baculoviridae* family [42,43]. Finally, nudiviruses were classified as members of a separate family, *Nudiviridae*. The members of *Nudiviridae* infect a broad range of hosts, from terrestrial to marine and freshwater hosts. In addition, nudiviruses are believed to appear in any place where insects and crustaceans occupy, resulting in the appearance of nudiviruses in all continents [15].

The name *Nudiviridae* originates from the lack of occlusion bodies in nudivirus [18]. However, occlusion bodies have been identified in some nudiviruses such as PmNV, ToNV and OrNV (facultatively occluded) [14,36, 44], suggesting that occlusion bodies are not a unique characteristic of baculoviruses alone.

Genomic studies revealed 31 core genes, including homologues from baculoviral core genes that are shared among nudiviruses [45,46]. A more recent study found that 28, instead of 31, core genes are known among nudiviruses [18]. Although most of the core genes in nudivirus have been identified, *p6.9* was not identified in several nudiviruses due to the algorithm of gene finder tools in the past. The *p6.9* gene, which is responsible for nucleocapsid packaging/assembly, was not identified in FrNV, FvNV, DhNV and PmNV per the NCBI database [17,36]. However, upon further analysis of the PmNV genome sequence, Bézier *et al.* detected *p6.9* genes (64 881–65 078) [45]. Recently, the *p6.9* gene was identified in HzNV-2 (position 24 375–24 127) [45], CcNV (position 72 007–72 231) and CmNV (position 45 460–45 651) [18] using a manual blast search. The reason for this could be that the prediction tools are not able to recognize the repetitive serine (S) and arginine (R) as part of the potential protein sequence [18]. In this study, by using the repetitive SR, p6.9 was identified in PvNV (49 039–49 239), FrNV (111 022–110 747), FvNV (109 492–109 220) and MrNuV (ORF51). We were not able to locate the *p6.9* in the DhNV genome. The unknown function gene *GbNV-gb51-like* gene was not identified in both PmNV and PvNV genomes, although Bateman *et al*. [18] found that ORF62 in the PmNV genome was a *gb51-*like gene.

Homologous regions play vital roles in baculoviruses including DNA replication [47], gene transcription [48] and possibly homologous recombination [49]. These regions are characterized by a modular structure consisting of direct repeats and palindrome sequences [49]. The homologous regions are different among species regarding length, sequence and copy number. In addition, several homologous regions have been reported to distribute along the genome of baculovirus [36]. So far, however, no homologous regions are identified in the nudivirus genome, which may cause the differences in genome replication between nudiviruses and baculoviruses [14]. Like other nudiviruses, the PvNV genome contained 13 directed repeat sequences, and none of them contained palindrome motif, suggesting that PvNV might have DNA replication machinery, which is different from baculoviruses.

The phylogenetic tree constructed from orthologue/core genes revealed that PvNV falls in the same clade as PmNV (Fig. 3) supported by a posterior probability of 100, and the orientation of core genes and orthologous genes was similar in PvNV and PmNV (Fig. 4). Therefore, we propose that PvNV, like other nudiviruses isolated from crustaceans, should be classified as a member of *Gammanudivirus* genus.

Apoptosis is a process that leads to the death of cells. While this process is important for the development of multi-cellular organisms, it also plays a role in responses to viral infection by causing infected cells to undergo a cell death programme [50]. However, viruses can develop inhibitors of apoptosis protein (IAP) to inhibit the host’s apoptosis process, resulting in the expediting of virus proliferation and dissemination [51]. The IAP molecules have been reported in several viruses, such as baculoviruses, entomopoxviruses, iridoviruses, nudiviruses and asfarviruses [52,53]. The IAP found in the baculoviral genome is believed to have evolved from their host cell homologue [51]. Since nudiviruses used to belong to *Baculoviridae*, it is possible that the IAPs from nudiviruses were also derived from their host. In this study, ORF88 from PvNV was similar to IAP from *P. monodon* and PmNV, suggesting that the IAP from PvNV evolved from its host. Although IAPs have been proven to help baculoviruses prevent the viral-induced apoptosis process [51,54], the function of IAPs in nudiviruses, particularly in PvNV, needs to be fully investigated.

Polyhedrin protein is the major component that forms occlusion bodies in all baculoviruses and some nudiviruses. The promoter of polyhedrin from AcMNPV has been widely used in recombinant protein expression using baculovirus expression vector systems (BEVSs) [55]. However, the productivity of BEVS is not so different from the ones that were originally developed [56]. Therefore, productivity improvement for BEVS is worth studying. There are several strategies to improve the productivity of BEVS, such as the modification of viral promoters [57] or searching for a new promoter, which is stronger than the traditional promoters [58,59]. Thus, searching for new promoters in different viral families is an interesting topic.

In baculoviruses, the *polyhedrin* gene is a very late expression gene showing predominant expression at the very late phase of infection [57]. The *polyhedrin* gene was annotated in some nudiviruses, such as PvNV, PmNV, TNV, CmNV, CcNV, KNV, OrNV and MNV. However, the polyhedrin in those nudiviruses has not been fully functioning. In addition, occlusion bodies playing a role in transmission have been observed in PvNV, PmNV and ToNV. Since PvNV and PmNV are nudiviruses infecting penaeid shrimp, it is possible that the occlusion bodies from PvNV are also made of polyhedrin protein. However, further studies need to be done to confirm this hypothesis. In addition, the promoters of *polyhedrin* in those nudiviruses have not been identified. In this study, 200 nts upstream of their start codon were used to identify the polyhedrin promoter using the NNPP server. The results showed that the promoters from tested nudiviruses contained consensus sequences of AxTxxxxAxxxTxTAxxAxTxxTTxxxTxTTxAT. In baculoviruses, the early promoter contains the TATA box followed by TATA[TA][TA][TA] or 20–40 nt with either one or two initiator motifs, CA[TG] or CGTGC. The late promoter contains the motif of [ATG]TAAG [45]. The promoter region of *polyhedrin* from PvNV was predicted to have a TATA box, but it was not followed by any sequences above, suggesting that the gene regulation in PvNV may be distinct from the baculoviral gene regulation system. In addition, the expression of very late genes such as *polyhedrin* in baculovirus depends on the A/T rich sequence located between TGTA (within the TSS) and the translation initiation site. This A/T rich sequence is defined as burst sequence (BS) [57]. Interestingly, the sequence downstream of TSS in the predicted promoter in PvNV-polyhedrin showed A/T rich in the proportion, suggesting that this sequence might play a similar role with BS in baculoviruses. However, further experiments need to be carried out to determine the function of this sequence in polyhedrin expression in PvNV.

In summary, we sequenced and annotated the full-length genome sequence of PvNV, the first viral pathogen reported in shrimp almost 45 years ago. The PvNV genome contains a circular double-stranded DNA containing 119 883 bp, which encodes 101 putative ORFs. Homologous analysis revealed that PvNV belongs to homologue groups with nudiviruses. The results from the homologous analysis were supported by core gene sequence data, which showed that the PvNV genome contained all known nudiviral core genes. Phylogenetic analysis revealed that PvNV falls in the clade of *Gammanudivirus*. Therefore, we propose to reassign PvNV as a new member of the family *Nudiviridae*. Finally, we computationally predicted the promoter of the *polyhedrin* gene in PvNV. However, further experiments need to be conducted to confirm the result from *in silico* analyses.

## Highlights

- The complete genome sequence of *Penaeus vannamei* nudivirus, also known as Baculovirus penaei (BP), was determined.
- BP genome contains a circular, dsDNA of 119 883 bp in length containing 101 ORFs.
- Phylogenetic analyses revealed BP clusters with *Penaeus monodon* nudivirus.
- We propose to reassign BP to the family *Nudiviridae* instead of *Baculoviridae*.

## Supplementary material

10.1099/mgen.0.001360 Uncited Supplementary Material 1.

## Abbreviations

ApNV: *Aratus pisonii* nudivirus
BEVSs: baculovirus expression vector systems
BP: Baculovirus penaei
BS: burst sequence
CcNV: *Crangon crangon* nudivirus
CmNV: *Carcinus maenas* nudivirus
CsNV: *Callinectes sapidus* nudivirus
DhNV: *Dikerogammarus haemobaphes* nudivirus
EHP: *Enterocytozoon hepatopenaei*
FpNV: *Faxonius propinquus* nudivirus
FrNV: *Faxonius rusticus* nudivirus
FvNV: *Faxonius virilis* nudivirus
GbNV: *Gryllus bimaculatus* nudivirus
HgNV: *Homarus gammarus* nudivirus
HzNV-2: Heliothis nudivirus 2
IAP: inhibitors of apoptosis protein
IHHNV: decapod penstylhamaparvovirus 1
IMNV: infectious myonecrosis virus
KNV: Kallithea virus
MBV: monodon baculovirus
ML: membranous labyrinth
MNV: Mauternbach virus
MrNuV: *Macrobrachium rosenbergii* nudivirus
NGS: next-generation sequencing
NHP-B: *Hepatobacter penaei*
NNPP: Neural Network Promoter Prediction
OrNV: *Oryctes rhinoceros* nudivirus
PL: post-larvae
PmNV: *Penaeus monodon* nudivirus
PvNV: *Penaeus vannamei* nudivirus
PvSNPV: *Penaeus vanname*i singly enveloped nuclear polyhedrosis virus
R: arginine
S: serine
TNV: Tomelloso virus
TSS: transcription start site
TSV: Taura syndrome virus
*Vp*_AHPND_: acute hepatopancreatic necrosis disease-causing *Vibrio parahaemolyticus*
WOAH: World Organization for Animal Health
WSSV: white spot syndrome virus
YHV: Yellow head virus genotype-1
YHV: Yellow head virus genotype-1
